# The Role of Motivation, Glucose and Self-Control in the Antisaccade Task

**DOI:** 10.1371/journal.pone.0122218

**Published:** 2015-03-31

**Authors:** Claire L. Kelly, Sandra I. Sünram-Lea, Trevor J. Crawford

**Affiliations:** Department of Psychology, Fylde College, Lancaster University, Lancaster, LA1 4YF, United Kingdom; Centre de Neuroscience Cognitive, FRANCE

## Abstract

Research shows that self-control is resource limited and there is a gradual weakening in consecutive self-control task performance akin to muscle fatigue. A body of evidence suggests that the resource is glucose and consuming glucose reduces this effect. This study examined the effect of glucose on performance in the antisaccade task - which requires self-control through generating a voluntary eye movement away from a target - following self-control exertion in the Stroop task. The effects of motivation and individual differences in self-control were also explored. In a double-blind design, 67 young healthy adults received a 25g glucose or inert placebo drink. Glucose did not enhance antisaccade performance following self-control exertion in the Stroop task. Motivation however, predicted performance on the antisaccade task; more specifically high motivation ameliorated performance decrements observed after initial self-control exertion. In addition, individuals with high levels of self-control performed better on certain aspects of the antisaccade task after administration of a glucose drink. The results of this study suggest that the antisaccade task might be a powerful paradigm, which could be used as a more objective measure of self-control. Moreover, the results indicate that level of motivation and individual differences in self-control should be taken into account when investigating deficiencies in self-control following prior exertion.

## Introduction

Self-control refers to the regulation of behaviour and inhibition of automatic/impulsive actions, for example when suppressing a powerful emotional response to a disturbing experience [1]. When exercised daily, this important psychological process [2] is associated with positive life outcomes, whilst poor self-control is linked to increased social adversity [3]

Self-control requires the conscious and effortful control of actions, and contrasts with more unconscious/automatic forms of cognition [4]. Self-control is also an executive function (EF); a higher-order cognitive process associated with the dorso-lateral-prefrontal cortex (dlPFC) [5]. The activation of the dlPFC during self-control has been well documented using functional-magnetic-resonance imaging (fMRI) [6], with reduced activation during weakened self-control [7].

Several studies have shown that individuals have difficulty completing two consecutive self-control tasks; the first is completed normally, but there is usually a temporary impairment in performance on the second [8]. The resource depletion theory [9] suggests that this impairment stems from a depletion of resources with self-control drawing on a limited supply [10]. The effects are specific to self-control tasks and not found in relatively automatic and effortless tasks [8, 11]. These observations have been strongly supported across over approximately 80 studies using various measures of self-control [3].

One influential line of research suggests that self-control is glucose dependent [1]. Glucose is essential for brain function, and is distributed and metabolized according to areas of activation. It has been suggested that the amount of cognitive effort needed to complete a task moderates the susceptibility to changes in glucose availability, i.e. one factor that appears to be of particular relevance is whether tasks are cognitively demanding [12; 13]. The energy cost for effortful, controlled or executive processes appears to be significantly higher than that for automatic or reflexive processes [14] Indeed, lowered peripheral glucose levels following performance of a cognitively demanding task have been reported [15; 16]. These results indicate that cognitively demanding tasks and in particular those relying on executive functions are sensitive to changes in glucose availability.

A series of experiments explored that relationship with evidence showing that acts of self-control use relatively large amounts of glucose and that glucose administration can improve self-control (see [14] for review). More specifically, it has been shown that blood glucose levels declined following self-control exertion in an incongruent, but not congruent, Stroop task [15]. Weak self-control was reported following a reduction in glucose levels, induced by a previous task, but administration of a glucose load counteracted this [8]. Similarly, selfish desires were more frequently suppressed and there was an increase in offers of support to others, following glucose ingestion [17]. This supports the idea that self-control is weakened when blood glucose levels are low.

### Challenges to the resource depletion theory

Research suggests that the role of motivation in moderating one’s temporary deficiency in self-control following prior exertion needs to be considered. This idea is captured by the resource allocation account [18], which proposes that high levels of motivation will modulate acts of self-control irrespective of whether or not the individual has been exposed to a previous self-control task. This account acknowledges that engaging in self-control requires glucose, but argues against the strong claims of the resource depletion theory that self-control per se is sufficient to produce a significant diminishment or depletion [18, 19]. The account posits that while engaging in a self-control task, glucose will be assigned based on one’s intrinsic level of motivation to complete that task. Thus resources will be allocated if one deems the task to be important [18]. Therefore impaired performance that has typically been documented in a second task of self-control might be associated with how willing or motivated one is to engage in a task rather than—as the resource depletion theory claims—solely dependent on whether or not there is a sufficient resource to complete the task. Self-report measurements have been recommended to determine whether task importance and interest (i.e. intrinsic motivation) can offset the temporary deficiency one observes in the second of two self-control tasks [18].

Recent findings support the resource allocation account and challenge claims made by the resource depletion theory that glucose is significantly depleted during self-control exertion. Molden and colleagues, for example reported no decline in blood glucose after a self-control task was completed [20]. Further, a re-analysis of Gailliot and colleagues research disputes the observation that blood glucose levels significantly are reduced following the application of self-control [19].

Additionally, Sanders and colleagues [21] recently reported that a glucose facilitation effect on self-control was detected after gurgling with (not ingesting) a glucose drink. This evidence suggests that replenishing glucose resources through glucose ingestion may not be necessary to improve self-control and that glucose might exert its beneficial effects on self-control performance in a more indirect way. For example, glucose itself may be motivational and indeed its presence in the mouth stimulates brain areas associated with reward [22]. Framing Sanders and colleagues [21] findings within the resource allocation account, glucose may provide one of several pathways to increase one’s level of motivation—alongside the offering of monetary incentives [23] and one having feelings of autonomy towards task engagement [24]—which could then ameliorate the temporary deficiency in performance observed in a second task following the completion of an initial self-control task [18].

Baumeister and colleagues e.g. [25] have recently proposed a revision of their original resource depletion theory by incorporating the resource allocation account [18]. They now propose that motivation would only moderate the level of engagement in a subsequent task of self-control in situations where there is an insufficient level of resources—due to prior self-control engagement—available for further self-control acts.

The aim of this study was to further investigate the role of glucose in self-control and to examine the potential moderating role of motivation. In particular, by monitoring blood glucose levels over time the current study attempted to explore whether any decline in self-control was indeed linked with a significant reduction in peripheral blood glucose availability. A glucose drink was also administered to examine whether relative to placebo, glucose would improve the powers of self-control following exertion in the Stroop task.

Previous research has predominately used behavioural measures of self-control and there is a lack of investigations that have used more objective measures of self-control. One well-established objective measure is the antisaccade task (AST) [26], in which an automatic saccadic eye-movement to the onset of a visual stimulus is suppressed, and a voluntary saccadic eye-movement is elicited away from the stimulus [27]. The AST correlates well with other self-control measures (e.g. the Stroop) [28], and involves the activation of the dlPFC and subcortical networks [27, 29]. In addition, it serves as an implicit indicator of self-control and error awareness, which is measured in terms of the frequency of spontaneous corrections that are generated following an incorrect saccade towards the target. The AST also yields temporal and spatial information, and has been directly linked to inhibitory networks in the brain [30], which could provide a reliable and objective “biomarker” for self-control [3].

In the prosaccade task (PST) the goal is to look directly towards the target, which requires no inhibitory control [31]. The AST, together with this control task, would determine whether glucose administration influenced the general properties of saccadic eye-movements or more specifically, only those under self-control. Prosaccade (PS) responses are 100ms faster and produce less errors than antisaccade (AS) responses [29, 32]. We predicted a task that demanded strong self-control would impair subsequent AS (but not PS) performance and that supplementary glucose and/or level of motivation would attenuate this.

The study also examined the potential moderating effects of individual differences in self-control on saccade performance. We predicted—based on previous research—that poor levels of self-control would be linked with a larger decline in AS (but not PS) performance following self-control exertion [33, 34].

Overall, evidence has shown that consecutive self-control tasks lead to weakened performance levels in these tasks, and that supplementary glucose helps to restore control [14]. However, recent evidence is beginning to challenge the role of glucose in self-control. Consequently, this study employed a frequently used initial ‘depletion’ task (the incongruent Stroop) [3] to determine whether a temporary resource deficiency was responsible for the decline in subsequent performance of a novel more objective measure of self-control (the AST) and whether a glucose drink would restore performance. Both measures of self-control that were employed—the Stroop task and AST—required one to exert self-control by responding to an external prompt, for example a signal or instruction rather than relying on self-control, generated based on one’s personal intentions or decisions [35]. The research also addressed whether levels of motivation and individual differences in self control moderate the effects of depletion.

## Materials and Methods

### Research design

To examine the effects of glucose (vs. placebo) administration on self-control performance, the experiment used a double-blind placebo controlled, 2 (drink condition: glucose, placebo) x 2 (saccade task: PS, AS) mixed-factorial design with repeated measures on the second factor. Participants were randomly assigned to drink condition.

### Participants

Seventy one undergraduate and postgraduate students of Lancaster University participated voluntarily or in exchange for course credit. Four were excluded from the analysis due to their AS performance indicating that they failed to fully understand the task instructions. This resulted in a final sample of 67 participants (48 female, 19 male) aged between 18 and 38 years of age (*M* age = 21.15 years). This sample size was based on the previous literature which have shown clear effects of glucose deficiency on self-control (see three studies reported in [8] which employed sample sizes of 16, 12, and 24). The sample size was chosen to be in excess of those used previously (see [8]). Further, a power analysis calculation was conducted based on previous findings, which indicated that with an alpha level of. 05, a sample size of 67 would be sufficiently highly powered (.78) according to Cohen’s standards [36]. Potential participants were excluded on grounds of the following: diabetes mellitus (and/or history of the illness); any glucose intolerances; pregnancy, lactating. Ethical approval was granted by the Lancaster University Ethics Committee and all participants completed a form detailing their written consent with the right to withdraw before commencing the study.

### Materials

#### Initial measure of self-control: Stroop task

A computerised version of a frequently used incongruent Stroop task (colour words and ink colours were mismatched), containing 135 colour words (red, blue, green, yellow or purple) was administered [37]. Each word was presented for 2,000 ms and participants were instructed to respond to the ink colour of the word as precisely and as rapidly as possible by pressing particular keys on a QWERTY keyboard (‘P’ for purple, ‘Y’ for yellow, ‘G’ for green, ‘B’ for blue and ‘R’ for red) and to say aloud the ink colour. For words presented in red ink only, the word itself was to be read rather than the ink colour.

#### Self- control scale [38]

The full 36-item self-control scale was administered to assess individual differences in self-control. Each item was rated using a 5-point-Likert scale (1 = ‘*not at all like me’* to 5 = ‘*very much like me*’) providing an overall score.

#### Intrinsic Motivation Inventory (IMI) [39, 40]

Responding to the resource allocation account and the need to consider motivation [18], participants’ self-reported level of intrinsic motivation was examined using a 36 item IMI. Responses were made on a 7-point-Likert scale ranging from ‘*not at all true’* to ‘*very true’*. Example statements, which required a response included, ‘I enjoyed doing this activity very much’, ‘This activity was fun to do’, ‘I did this activity because I wanted to’ and ‘I would be willing to do this again because it has some value to me’. The items were summated to give an indication of how motivated participants were to complete the tasks, which they performed [41].

#### Saccade tasks [26]

Participants were seated with their head positioned on a chin rest located 57 cm from a 19″ monitor on which the visual task was displayed. An Eyelink 1000 (SR Research: 1,000 Hz, <.5° accuracy) was used to record response times of saccades towards the target. In both tasks, a fixation cross appeared in the centre of the screen. After 1,000 ms, a small green circle (.6° diameter) appeared 8° to the left or to the right of the cross. The target and cross remained on the screen for 1,000 ms, and a 1,500-ms interval preceded the next trial. The location of the target was randomised and appeared on the left or right with equal frequency. Participants were instructed to direct their eyes towards the circle (PST) or away—and to the opposite side of the circle (AST) as quickly and as accurately as possible. Two specific saccade parameters—reaction times (latencies in milliseconds; ms) and the frequency (%) of directional errors—were measured in order to compare potential performance differences in the PST and AST.

#### Blood glucose levels

Measured at baseline (time 1), after Stroop task completion (time 2) and after both the PST and the AST were administered (time 3) with the Exactech measuring equipment (supplied by Medisense Britain Ltd), according to the manufacturers’ guidelines. Akin to those used by individuals with diabetes mellitus, this portable finger-prick glucose monitoring device provides a reliable measure of blood glucose [42]. Further, a recent study found a strong correlation between capillary blood glucose measures (finger prick) and AV blood [43], confirming its measurement sensitivity.

#### Sensory Evaluation Form [44]

Participants rated how much they liked the drink across five categories on a 9-point Likert measure (1 = ‘*like extremely’* to 9 = ‘*dislike extremely’*).

#### Drinks

A glucose or inert placebo drink, matched for sweetness and flavoured with lemon juice were randomly administered in transparent plastic cups, at room temperature, following a double-blind procedure (see Table 1 for drink compositions). Sensory evaluation revealed that the glucose and placebo drinks were liked equally (F (1, 66) = .59, p = .45).

**Table 1:** Drink composition.

Glucose	Placebo
25g glucose (dextrose) powder (Thornton & Ross Ltd, Huddersfield, HD7 5QH), 10ml lemon juice, 250ml cold water	5 Saccharin tablets (Sweetex, Reckitt Benckiser PLC, Slough, SL1 3UK, UK), 10ml lemon juice, 250ml cold water

### Procedure

Each participant attended one testing session (approximately 50 minutes). Prior to testing, participants were provided with information about the study and asked not to consume anything except water for at least two hours and refrain from consuming any alcohol for at least 12 hours before the session.

On arrival, participants were given an information sheet and gave informed consent and demographic information. For each task, full instructions were provided. At the beginning of testing, participants’ baseline blood glucose levels were measured (time 1). Participants then completed the computerised Stroop task (which lasted approximately four minutes, thirty three seconds) followed by a second blood glucose reading (time 2) and the double-blind administration of the glucose/placebo drinks. Participants evaluated the overall likeness for the drink using the sensory-evaluation form [44] and then completed the self-control questionnaire [38]. Participants were then seated in front of the eye-tracking computer and the headset and saccade tasks were prepared. Fifteen minutes following drink administration (allowing for glucose absorption), the saccade tasks were conducted over an approximate 6 minute period. During this time participants were instructed to remain as still and as relaxed as possible to ensure that accurate readings were taken. The PST was administered first, followed by the AST, due to evidence of carry over effects from the AST [45]. A final blood glucose reading was recorded (time 3) after completing the saccade tasks. The IMI [39; 40] was then administered before participants were fully debriefed.

### Statistical analysis

Saccade latencies (saccade reaction times; RTs) were computed as the delay between target onset and the start of the first saccade, with an amplitude of greater than 2°. Responses of less than 80 ms or over 500 ms were regarded as anticipatory or late saccades, respectively, and were excluded from analysis. For directional errors, the total number of errors (incorrect saccades made towards rather than away from the target) in the AST was obtained and then converted to a percentage error rate based on the number of trials completed for each participant.

Initially, a correlational analysis on the overall data was conducted. Following this, using IBM SPSS Statistics Version 20, saccade performance was analysed using mixed-effects modelling. Specifically, we ran through a series of separate models for the two measures of performance in the saccade tasks; reaction times (RTs) and AST directional error rate (with F values drawn from ‘*Type III Tests of Fixed Effects’*). In the models, participants were included as random effects and task type for saccade RTs as well as drink condition were included as fixed effects. Self-reported levels of both motivation and self-control were added as covariates to the models to assess whether differences in motivation and self-control moderated drink effects on saccade performance (reaction times and/or directional errors) or whether they were significant predictors of performance irrespective of drink condition. Similarly, glycaemic response was analysed using mixed effects modelling, again treating participants as random effects and both drink condition and time (T1: baseline, T2: post Stroop task completion and T3: post drink and saccade task performance) as fixed effects. Self-reported levels of motivation were also added as a covariate to the model to assess whether differences in motivation was a significant predictor of glycaemic response.

## Results

### Correlations

As demonstrated in Table 2, the correlational analyses revealed a significant negative relationship between motivation and directional error rate in the antisaccade task; those high in motivation produced fewer errors in the AST. Additionally PS RTs were significantly positively correlated with AS RTs; longer responses in the PST were paired with longer response times in the AST. Further, the correlation analysis revealed a negative relationship between PS RTs and error rate in the AST; faster PS responses were paired with more erroneous responses in the AST.

**Table 2:** Correlation matrix.

Variable	1	2	3	4	5
1.Motivation	——	.123	.005	-.220	-.385**
2. Self-control		——	.144	.071	.042
3. Prosaccade RT			——	.325**	-.252*
4. Antisaccade RT				——	.110
5. Antisaccade directional error rate					——

*Note*. ** p <. 01,

* p <. 05, RT = response times

### Drink condition effects (glucose vs placebo)

#### Blood glucose levels

**Fig 1:**
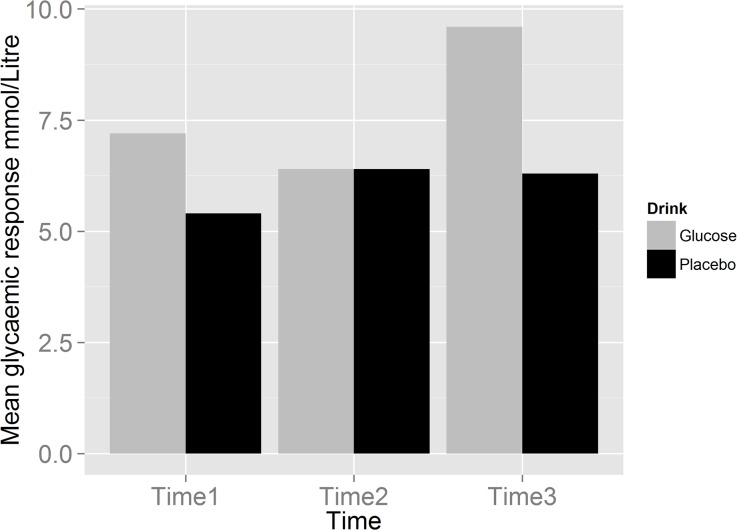
Mean (±95% confidence intervals) blood glucose level measurements over time as a function of drink condition.

There was a significant effect of time (F (2, 71.59) = 52.99, p = .00) and of drink condition (F (1, 68.46) = 64.10, p = .00) and a significant time x drink condition interaction (F (2, 71.86) = 49.32; p = .00). Further post-hoc analyses for the interaction revealed that following glucose administration (time 3), blood glucose levels were significantly higher compared to times 1 and 2 (p = .00) and all blood glucose levels following placebo (all p<.01; see Fig 1 created in R, Version 0.98.501). No significant differences in blood glucose levels over time were detected following placebo administration. Specifically, no significant reduction in blood glucose levels from baseline was observed after Stroop task completion (time 2; p = .96).

#### Saccade Performance

Initially the model was fitted to account for both participants as random effects and task type as a fixed effect. This analysis revealed a significant relationship between saccade RTs and task type (*β* = 63.13, t (66) = 11.61, p <. 001). There was a significant effect of saccade condition on RTs (F (1, 67) = 136.88, p = .00); PS RTs (*M* = 200.99, *SD* = 36.72) were significantly faster than AS RTs (*M* = 264.13, *SD* = 39.76). We then added drink condition and the interaction between drink and task type as fixed effects to the model. There was no effect of drink condition (F (1, 67) = 0.38, p = .54) or a task x drink condition interaction (F (1, 67) = 0.80, p = .37) on saccade RTs. Moreover, for directional errors, the initial model with drink condition as a fixed effect and participants as random effects showed drink condition to not be a significant predictor of AS directional error rate. (F (1, 65) = .00, p = .96).

### Intrinsic Motivation and Individual differences in self-control reported using the self-control scale [32]

**Fig 2:**
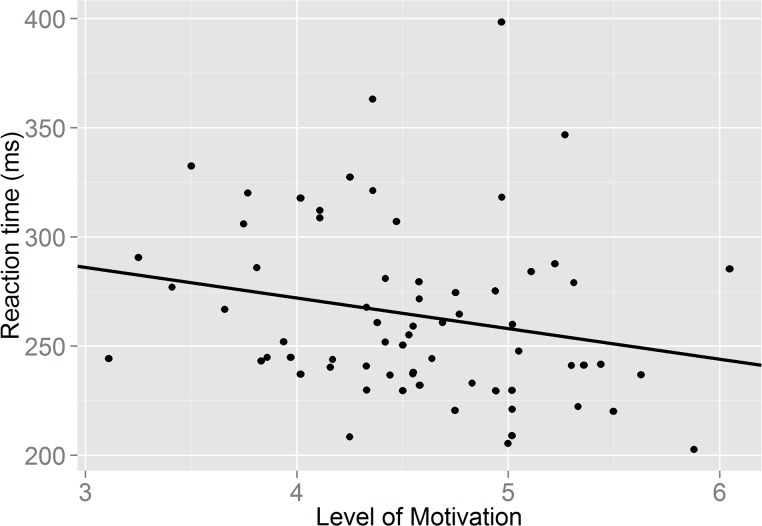
A graph showing the relationship between self-reported level of motivation and speed of responding in the antisaccade task.

We initially checked whether there were differences in motivation and self-control by drink condition. There was no significant differences in levels of motivation following the glucose and placebo drinks (F (1, 66) = .04, p = .84); nor were there any significant differences in reported self-control between groups (F (1, 66) = .56, p = .46). Adding motivation and self-control to the model did not result in significant drink effects on saccade RTs (F (1, 67) = 0.63, p = .80) or directional errors (F (1, 63) = .002; p = .96). Moreover, the task x motivation interaction failed to reach significance for saccade RTs, (F (1, 67) = 2.92; p = .09) and further inspection revealed that higher motivation did not significantly predict AS RTs (*β* = -14.49, t (65) = -1.65; p = .10) (see Fig 2; created in R, Version 0.98.501). In addition no significant task x self-control interaction was observed on saccade RTs (F (1, 65) = .20, p = .65).

**Fig 3:**
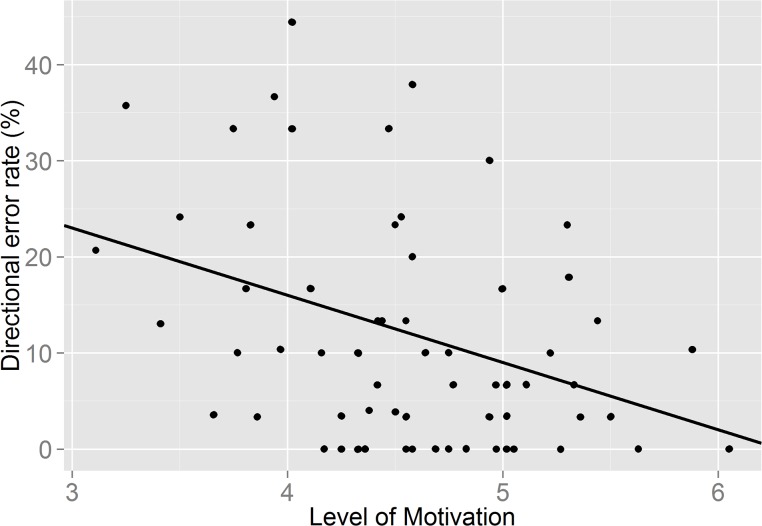
A graph showing the relationship between self-reported level of motivation and directional error rate in the antisaccade task.

However, interestingly adding motivation to the model showed that motivation itself was a significant predictor of frequency of directional errors, irrespective of drink condition (F (1, 63) = 11.53; p = .001) The relationship between directional error rate and motivation was negative (*β* = - 7.42, t (63) = - 3.395, p = .001), indicating that higher motivation resulted in fewer errors being committed in the AST (see Fig 3; created in R, Version 0.98.501). These results show that when taking into account the amount of self-reported motivation to perform the second self-control task, the type of drink consumed does not become a significant predictor of task performance. However, motivation itself appears to be a better predictor of task performance. Individual differences in self-control did not predict errors on the antisaccade task (F (1, 61) = .581, p = .449), however there was a significant drink x self-control interaction (F (1, 61) = 4.074; p = .048). For those consuming the glucose drink, levels of intrinsic self-control predicted frequency on the number of directional errors, with higher levels of self-control resulting in fewer errors (*β* = -.308, t (61) = -2.018; p = .04).

We also evaluated the effect of motivation on blood glucose levels, as these might be affected by level of motivation due to potential differences in neuroendocrine activation. However, addition of motivation as a covariate to the model did not change the previously observed main effect or interaction. In addition, motivation itself did not significantly predict blood glucose levels (F (1, 63.48) = 1.66; p = .20).

## Discussion

The resource depletion theory argues that self-control is resource limited and has been supported with evidence that two successive self-control tasks yield a decline in self-control in the second task [1; 3]. It has been suggested that glucose is the major resource for self-control and that administration of a glucose load can reduce the temporary deficiency in performance in a second task of self-control [8]. In the current study administration of a glucose drink was expected to enhance performance in the AST in comparison to placebo following self-control exertion in the Stroop task. This prediction was not supported, and therefore failed to replicate previous research e.g. [8, 17].

However, our findings revealed a strong effect of motivation on self-control. Level of motivation was a significant predictor of directional errors in the AST, suggesting that higher levels of self-reported motivation were related to the production of fewer errors in the task. However, low levels of self-reported motivation resulted in a greater number of directional errors in the AST, which is indicative of self-control failure. These observations provide support for the resource allocation account [18], as low levels of self-reported motivation to complete the AST produced the expected deficiency in self-control performance—greater errors—following prior engagement of self-control in the Stroop task. Further, although there was a trend to suggest that level of motivation was a predictor of AST response speed, which is supportive of the resource allocation account that motivation has an ameliorating effect on the temporary deficiency in subsequent self-control performance [18], this observation was not significant.

Previous work [31] has shown, faster saccadic reaction times are associated with a higher AST error rate. In the current study, the weak correlation between AS reaction times and motivation suggests that the relationship between motivation and directional error rate is not mediated by effects on reaction time. These data would therefore imply that motivation is not exerting its effect on error rate via this route, but that motivation might be having a more direct effect on AS error rates. This provides stronger supportive evidence for the role of motivation in alleviating the temporary deficiency in AST performance following prior self-control exertion.

No effect of motivation levels were observed in the PST, which required no inhibitory control, suggesting that prior self-control exertion did not influence the general properties of saccades. It is worth noting that previous research has found that the effects a self-control task has on subsequent exertion are quite specific. Tasks pertaining to self-control, not general cognitive measures, are diminished following self-control exertion [11]. Here, motivation level did not significantly predict performance outcome in the PST, this finding was thus unique to the AST, which required effortful cognitive control.

In line with previous research e.g. [20; 21] no significant decline in peripheral glucose levels was observed after the Stroop task. It has previously been argued that the failure to observe a drop in peripheral glucose levels after performing a self-control task challenges the resource component of the resource depletion theory, specifically the notion that an energy substrate like glucose is depleted during self-control exertion [19]. However, it is important to note that the failure to observe a decline in peripheral blood glucose levels does not necessarily mean that there is no temporary shortage in energy supply centrally. For one, it has been argued that the glucose measuring devices used in this study and previous research (e.g. [15; 8]) may lack sensitivity to detect such subtle reductions in glucose levels [18; 19]. Moreover, changes in peripheral levels do not necessarily equate to changes in the level of glucose in the brain [19].

Although evidence suggests that brain glucose levels are approximately 15–20% of blood levels [46; 47; 48; 49; 50], glucose metabolism varies throughout tissue and cell type in the brain. Both the rate of blood to brain glucose transport [51] and glucose metabolism [52] are stimulated in different areas in the brain during cognitive tasks relevant to that area. There is disagreement about the additional energy costs associated with task performance, ranging from as little as 0.5% to 1.0% of the total energy budget [53] to evidence suggesting that performing cognitively demanding tasks increases total brain consumption by as much as 12% [54]. However, regardless of the actual level of additional cost, it takes approximately four to six seconds following neural activation for blood flow to increase, which suggests a temporary energy shortage in neurons may occur [53]. This (temporary) insufficiency has been suggested to underlie the improvement effect of glucose ingestion upon cognition [55]. Microdialysis measurements of brain glucose have shown a large decrease in hippocampal extra cellular fluid (ECF; 32 ± 2%) in rats tested for spontaneous alternation on a four-arm maze (a difficult memory task), while a smaller decrease (11 ± 2%) was seen in rats tested on a simpler three arm- maze, suggesting that the changes observed in ECF glucose are related to task difficulty. Moreover, there is some evidence that the concentration of ECF glucose in the brain after its transfer across the blood-brain barrier from plasma glucose varies with brain region (for review, see [56]). The cerebral cortex has comparatively low stores of glycogen (5–6 mmol/l compared to for example 13 mmol/l in the hippocampus [57]) suggesting that it might be particularly sensitive to those temporary deficits. Consequently, the failure to observe a significant decline in peripheral blood glucose levels after the initial self-control task [20], does not necessarily imply that there are no slight alterations in glucose levels following completion [19] and thus that exerting self-control does not rely on the availability of glucose.

Although we observed a powerful effect of motivation and reported no effect of glucose on self-control performance this also does not mean that the limited resource view of self-control should be dismissed. What these observations do reinforce however, are trends that have recently emerged in the self-control literature, i.e., that a reconsideration of the resource model is perhaps needed [19] with additional factors taken into account. As mentioned earlier, Baumeister and colleagues [25] have suggested possible amendments to the resource depletion theory, which acknowledges the resource allocation account [18] and the moderating effects of motivation. In particular it has been argued that motivation can counteract the temporary deficiency in self-control following prior exertion when glucose resource levels are low [25].

The current findings together with previous evidence therefore suggest that motivation might be an important factor in the temporary deficiency in self-control one observes in a subsequent task following exertion. More broadly, self-control performance and more specifically the degree to which it is affected by glucose availability, appears to be influenced by individuals’ motivation to exert self-control.

According to one interpretation, glucose might be motivational, thus stimulating reward areas in the brain [22]. For example self-control performance improved for participants that simply gurgled with glucose (not placebo) before a second task of self-control was completed [21]. The current study did not detect such an effect on motivation with the glucose drink and supports more recent evidence which reported no effect of glucose whether swilled or digested on self-control ability after prior exertion [58]. The neurobiological mechanisms underlying the observed moderating effects of motivation need to be explored. However, it is feasible to speculate that the motivation reported by participants may result or indicate a brief acute stress response mediated by sympatho-adrenomedullary axis (SAM axis) activation. Indeed, it has been argued that the importance of a task determines the initial preparedness via activation of one or both major endocrine systems, the hypothalamic-anterior pituitary-adrenocortical axis (HPA) and the SAM axis [59]. A major physiological role of activation of both endocrine systems is considered to be a temporary increase in energy production and more specifically provision of additional metabolic fuel through increase in glucose availability [60]. Consequently, in physiological terms motivation to perform a task, could lead to an intrinsic rise in glucose availability which in turn ameliorates any potential energy shortage. In the current study, motivation did not appear to change blood glucose levels. However, as these changes would arguably be very small, these might not have been detected due to lack of sensitivity of the measuring device. Alternatively, differences in motivation might affect performance through selective allocation of available resources; i.e. resources are channelled to areas of the brain pertaining to tasks that are seen as sufficiently important.

Another interesting finding emerged from analysis of the moderating effects of individual differences in self-control on subsequent performance. Individuals higher in self-reported levels of self-control performed the AST with greater accuracy following glucose consumption than those lower in self-control. This is interesting and requires further exploration, as it is somewhat incongruent with the resource depletion account. In particular one would expect that those low in self-control to be more susceptible to consumption of glucose than those high in self-control due to a greater vulnerability to resource depletion [3]. In the current study the opposite was observed.

This is the first study to investigate the effect of completing an initial self-control task on performance in the AST. The results of our study suggest the AST as a promising objective task of self-control, which can be simply administered [29] and compared to previous measures, assesses self-regulation on an implicit level by recording saccades that correct directional errors. It also uses the single modality of vision for both processing the stimulus and the behavioural response, in contrast to previous methodologies, where the stimulus was encoded for example visually, but an auditory or motor response was required. This modality task switching limited the extent to which self-control could previously be directly assessed [61].

Consequently, the findings have helped to address a particular problem inherent in the existing research field on self-control, which is the current difficulty in finding congruency in the measurement of self-control [3]. A wide range of tasks have been used to study the effects of a temporary deficiency in self-control ability [62]. Moreover, evidence linking glucose administration as well as the moderating effects of motivation and self-control to cognitive functions associated with restraint and willpower have not been validated using “biomarkers” of self-control. The results of this study demonstrated that implementation of the more objective AST as a measure of self-control within a self-control depletion paradigm is feasible.

Although our observations are reflective of more recent studies, it is important to note that in comparison to previous studies on self-control, the dose of glucose administered in the current study was lower (25g) compared to studies which showed beneficial effects with larger (35-40g) doses (e.g. in [8] and [17]). The effect of glucose administration on cognitive tasks, including memory follows an inverted U-shaped dose response curve [63; 64]. Although, the dose we used has previously shown to be optimal for facilitation of memory performance, it might be the case that a higher dose is needed to facilitate tasks pertaining to frontal lobe functions. Thus, further investigations are clearly required to determine the dose-response relationship between glucose, self-control performance and levels of motivation.

A further limitation of the current study that needs to be addressed by future research would be the more specific assessment of the ameliorating effects of motivation on self-control performance. This could be done by manipulating levels of task motivation rather than recording overall task motivation.

### Conclusion

In summary, the results of the current study suggest the AST could be a potential ‘biomarker’ of self-control and thus a powerful paradigm to use in future studies on self-control. Moreover, the study did provide further support for the resource allocation model, as motivation—specifically, how personally relevant it was to participants to complete the task—influenced self-control performance more powerfully than glucose administration. More specifically, higher motivation appeared to ameliorate the temporary deficiency in a self-control task, as participants with low motivation produced a weaker AS performance following self-control exertion. More research is needed to explore the role of factors (including motivation and self-control) that can act as response modifiers and neuroendocrine mechanisms should also be evaluated as a potential source for variability.
